# Une cause rare de plexopathie brachiale: une metastase d'un cancer du sein

**DOI:** 10.11604/pamj.2014.18.12.1008

**Published:** 2014-05-03

**Authors:** Mustapha Maâroufi, Imane Kamaoui, Meriem Boubbou, Nadia Sqalli, Siham Tizniti

**Affiliations:** 1 Service de Radiologie, Centre Hospitalier Universitaire Hassan II, Fès, Maroc

**Keywords:** Plexus brachial, IRM, cancer du sein, métastase, brachial plexus, MRI, breast cancer, metastasis

## Abstract

Nous rapportons le cas d'une patiente de 50 ans ayant une histoire de cancer du sein et qui accuse une symptomatologie d'atteinte du plexus brachial. L'IRM montre une masse qui envahie le plexus brachial compatible avec une métastase. L'IRM est très utile pour le diagnostic et l'orientation thérapeutique des plexopathies brachiales chez les femmes présentant un cancer du sein

## Introduction

Le plexus brachial est un ensemble de structures nerveuses situées de part et d'autre de la colonne cervicale, de localisation superficielle assurant l'innervation sensitivomotrice des membres supérieurs. Les pathologies du plexus brachial sont des pathologies peu connues, souvent mal diagnostiquées. La pathologie tumorale est la deuxième en fréquence après la pathologie traumatique, représentée essentiellement par l'envahissement par une tumeur de l'apex pulmonaire. Les envahissements métastatiques sont plus rares et sont souvent l'apanage du cancer du sein, c'est ce que nous allons discuter à travers ce cas clinique.

## Patient et observation

**Figure 1:**
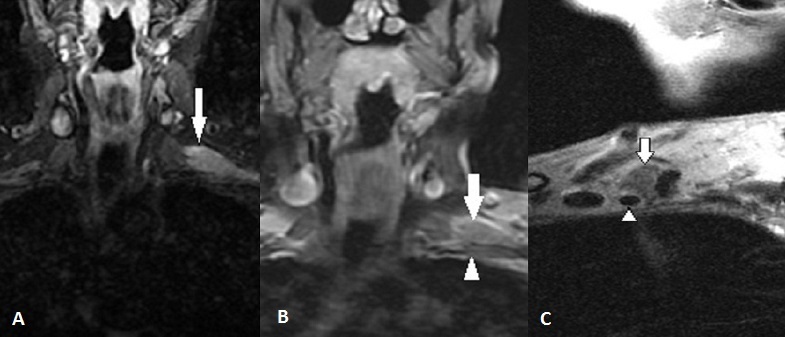
IRM en séquence STIR (A) et T2 (B) en coupe coronale et en séquence T2 en coupe sagittale (C): masse sus claviculaire gauche (flèche) de limites finement irrégulières en hypersignal STIR et en hypersignal modéré T2 au contact de l'artère axillaire (tète de flèche) et envahissant les faisseaux du plexus brachial

Il s'agit d'une femme de 50 ans, suivie depuis quatre ans pour une tumeur du sein gauche pour laquelle elle a bénéficié d'une mastectomie de type Patey avec curage ganglionnaire axillaire suivie d'une radiothérapie d'une dose globale de 40Gy, elle accuse depuis 3 mois des paresthésies et une lourdeur du membre supérieur gauche avec à l'examen clinique un déficit moteur proximal et distal des fléchisseurs et des extenseurs, une abolition des reflexes ostéo-tendineux bicipital et tricipital, une hypoesthésie de tout le membre et une amyotrophie de la main. L'Electromyogramme trouve une abolition des réponses motrices et sensitives de l'ensemble des nerfs du membre supérieur. L'IRM du plexus brachial (Figure 1) révèle une lésion nodulaire sus claviculaire de forme arrondie et de contours irréguliers envahissant les troncs plexiques. L'aspect IRM est équivoque de métastase envahissant le plexus brachial.

## Discussion

Chez les patientes traitées pour cancer du sein, l'extension métastatique et les lésions post-radiques sont les étiologies les plus communes des plexopathies brachiales. Sur le plan clinique, 85% des ces patientes se présentent pour des douleurs de l’épaule et du bras avec souvent une distribution en dermatome, et 15% se présentent pour des paresthésies. L'examen clinique révèle un déficit moteur, une atrophie musculaire et une hypoesthésie dans 75% des cas. Le syndrome de Claude Bernard Horner survient dans 50% des cas [1].

En raison du drainage lymphatique majoritaire du sein via le creux axillaire et sus claviculaire, l'envahissement métastatique du plexus brachial en contexte de cancer du sein n'est pas rare. Les adénopathies tumorales infiltrent les troncs et les faisceaux nerveux au niveau du creux sus claviculaire et plus distalement les nerfs au niveau de leur passage axillaire. L'envahissement des éléments vasculaires de contigüité est également possible [2].

L'IRM est actuellement le moyen le plus pertinent pour l’étude du plexus brachial et offre de nombreux avantages en comparaison avec le scanner. En effet, le caractére multiplanaire et la haute résolution en contraste permettent la différenciation des éléments nerveux des vaisseaux et des autres tissus avoisinants [3]. Les métastases présentent l'aspect de masse en hyposignal en T1 et en hypersignal modéré en T2 (par rapport au muscle). Le rehaussement est en général modéré et l'augmentation de volume tumoral détermine un aspect hétérogène en raison des phénomènes de nécrose [4, 5, 6]. Chez les femmes ayant subi une radiothérapie axillaire voire sus claviculaire, la distinction entre une métastase et une neuropathie radio-induite peut être difficile. Les lésions neurologiques après radiothérapie peuvent apparaitre après un délai de plusieurs mois voire années et semblent plus fréquentes après une dose totale dépassant 60Gy [7]. La traduction en IRM de la fibrose post-radique du plexus brachial se manifeste par un épaississement diffus avec rehaussement du plexus brachial sans masse focale et des anomalies de signal de la graisse avec un hyposignal T1 et T2 similaire au muscle. Le signal en T2 peut aider dans la différenciation de la fibrose post-radique de l'Infiltration tumorale précoce sans véritable effet de masse. En effet, la fibrose post radique est en hyposignal T2 alors que le tissu tumoral est en hypersignal T2 modéré. Parfois la discrimination entre métastase et fibrose post-radique est impossible en IRM, le recours se fait alors à la tomographie par émission de positron PET FDG qui permet de confirmer le diagnostic de métastase en montrant une hypercaptation du traceur au niveau du site lésionnel [8].

## Conclusion

L'IRM reste le moyen d'imagerie de première intension devant un tableau clinique d'atteinte du plexus brachial. Le diagnostic de métastase quoiqu'il est rare doit être systématiquement envisagé devant toute anomalie plexique rencontrée chez une patiente suivie pour cancer du sein. Elle permet dans la quasi-totalité des cas d'en faire le diagnostic et d'en orienter le traitement.
